# A clinical study of the piezosurgery, high-speed contra-angle handpiece, and the combined application of both for extraction of embedded supernumerary teeth

**DOI:** 10.1186/s12903-023-02829-y

**Published:** 2023-03-12

**Authors:** Maihepireti Maihemaiti, Ailimaierdan Ainiwaer, Ling Wang

**Affiliations:** 1 Outpatient Department of Oral Surgery, The First Affiliated Hospital of Xinjiang Medical University (Affiliated Stomatological Hospital), Ürümqi, Xinjiang China; 2 Research Institute of Stomatology of Xinjiang Uygur Autonomous Region, Ürümqi, Xinjiang 830054 China

**Keywords:** Extraction, Embedded supernumerary teeth, Piezosurgery, HSCAH

## Abstract

**Objectives:**

To compare the effectiveness of three methods: high-speed contra-angle handpiece (HSCAH), piezosurgery, and combined in the extraction of different locations and types of embedded supernumerary teeth.

**Methods:**

Sixty cases with different locations and different types of embedded supernumerary teeth were randomly divided into three groups for extraction by HSCAH, piezosurgery, and the combination of both, and the intraoperative and postoperative conditions of the three groups were compared and analyzed.

**Results:**

In the extraction of embedded supernumerary teeth in the inverted, horizontal, and root tip positions, the piezosurgery group required significantly longer operative time and reduced intraoperative bleeding compared with the HSCAH and the piezosurgery combined with the HSCAH; it could effectively relieve postoperative pain and facial swelling. In the extraction of oblique, orthodontic, middle, and crown segments of embedded supernumerary teeth, the use of a piezosurgery combined with an HSCAH can effectively reduce the operative time, while the factors of bleeding, postoperative pain, and facial swelling not statistically significant when compared with a piezosurgery. Compared with the HSCAH and combined piezosurgery, piezosurgery can significantly reduce the fear of patients.

**Conclusion:**

Piezosurgery is effective in extracting embedded supernumerary teeth in inverted, horizontal, and apical positions, effectively reducing intraoperative and postoperative trauma and shortening the time required for healing. The piezosurgery combined with an HSCAH can effectively reduce intraoperative and postoperative trauma when extracting embedded supernumerary teeth in oblique, orthodontic, middle, and crown positions. piezosurgery is a technique suitable for the treatment of patients with fear.

## Introduction

During the growth and development period, abnormalities in tooth structure, morphology, number, and eruption occur as a result of systemic or local factors. Multiple teeth, also known as extra teeth, are teeth that exceed the normal number of teeth and are numerically abnormal developmental anomalies [1]. The mechanism of the occurrence of multiple dentitions is still unclear, but some scholars have identified several factors associated with the occurrence of multiple dentitions such as dichotomization of the tooth buds; overactive or broken tooth plates; reversion phenomenon; abnormal developmental syndrome-like diseases; genetic and environmental factors [2].

According to the state of eruption, multiple teeth can be divided into eruption and ambush type, about 75% of the multiple teeth have been ambushed, and the rest of the extra teeth is part or all eruption [3]. The harm to oral health is the embedded type of supernumerary teeth. Usually cause the delayed eruption of permanent teeth, dislocation eruption, dental column gap being too large or crowded, and other dental occlusion disorders, serious and even lead to cranial and maxillofacial deformity [4]. Some ambush more teeth compression adjacent root to cause adjacent root shape absorption. Some investigators believe that supernumerary teeth are even associated with odontogenic cysts and tumor development [5]. Therefore, when this adverse oral damage occurs, we should timely remove extra teeth as soon as possible. Ambush multiple teeth have diverse forms, and complex anatomical positions, and are closely related to the important tissue structure of the maxillary sinus, nasal base, anterior nasal spine, and nasal and palatal nerve [6]. Surgery is difficult to remove, so the choice of surgical tools and instruments is particularly important. In the past, bone hammers, bone chisels, and other instruments were used to drive the teeth, which will cause serious postoperative complications and bring great pain to patients [7, 8]. With the continuous development of surgical techniques, minimally invasive tooth extraction instruments are gradually born and applied in clinical practice. It has been found that piezosurgery has a selective cutting function, stopping cutting when adjacent to nerves and blood vessels during osteotomy, effectively protecting soft tissues such as nerves and blood vessels. However, slower bone cutting and distraction thing prolonged [9]. HSCAH can cut bone and tooth separation faster, which can effectively shorten the operation time. However, because of the too-fast cutting speed, it is easy to lead to damage to the adjacent soft and hard tissue [10].

This paper compares different schemes, including piezosurgery, HSCAH, and combined ultrasound knife, and compares the operation time, intraoperative bleeding amount, postoperative pain, facial swelling, and fear. It provides certain theories and data for clinical application. Details are as follows.

## Materials and methods

### General information

**Fig. 1:**
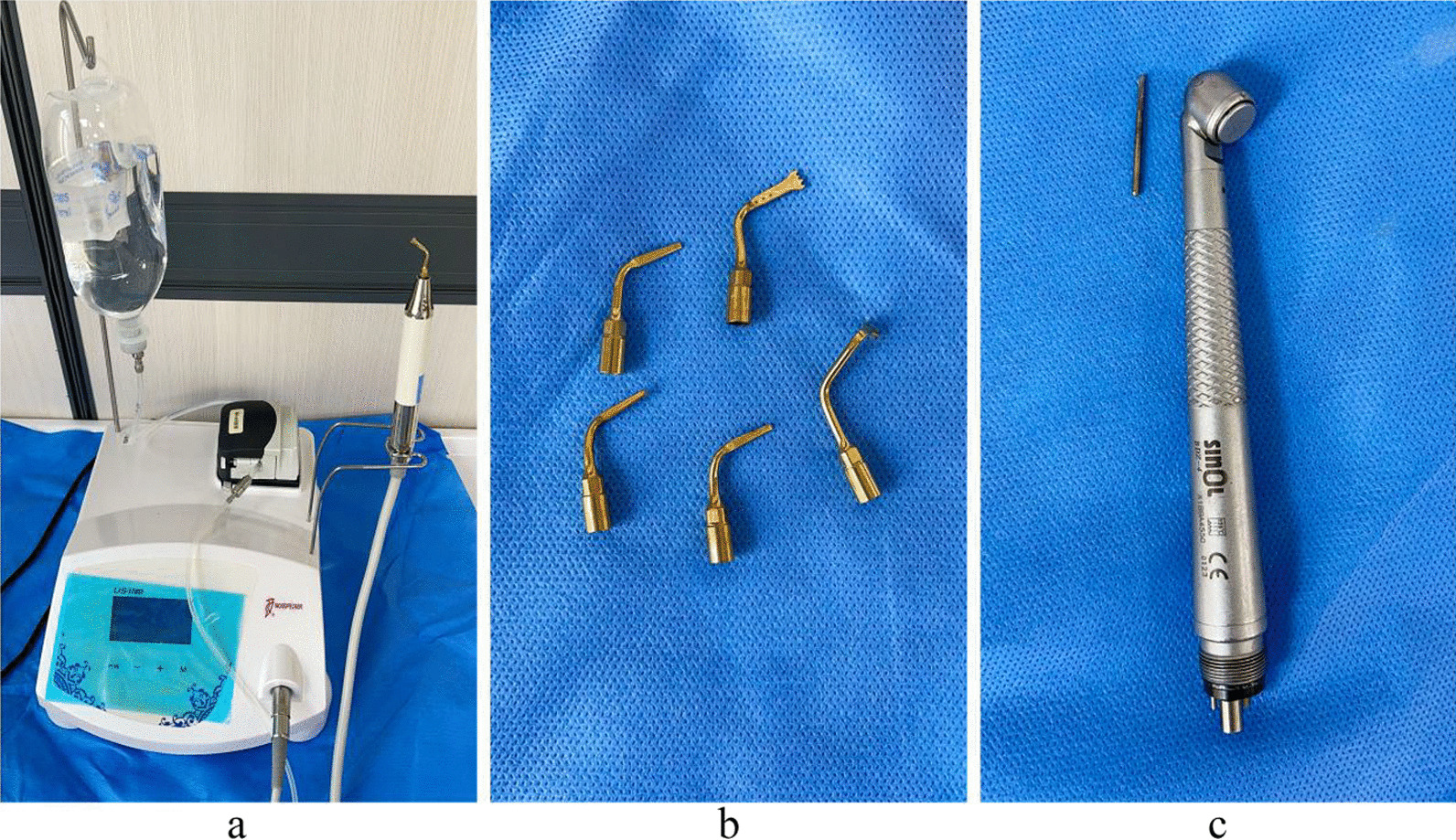
**a** piezosurgery; **b** piezosurgery cutter head; **c** HSCAH and cleavage drill

Sixty patients with a total of 60 completely bone-embedded supernumerary teeth were selected from December 2021 to May 2022 who attended the maxillofacial surgery clinic of the First Affiliated Hospital of Xinjiang Medical University, of which 32 were male and 28 were female, aged 32 years, with an average age of (10.93 ± 7.192) years. 10 cases (16.7%) were orthotropic embedded supernumerary teeth; 14 cases (23.3%) were oblique; 24 cases (40%) were inverted; 12 cases (20%) were horizontal. All 60 patients had no contraindications to extraction and voluntarily participated in the study and signed the informed consent form. 60 patients were randomly divided into three groups, the first group applied the piezosurgery method for tooth extraction, the second group used HSCAH, and the third group piezosurgery combined with HSCAH (Fig. 1a–c). The general data of the patients in the three groups were compared, and the differences were not statistically significant (*p* > 0.05) and were comparable, Table 1. The study was reviewed and approved for implementation by the ethics committee of the author's hospital.

**Table 1:** General information

	Piezosurgery	HSCAH	Combined application of both	Statistics	*p*
*Gender*					
Male	11 (55.0%)	12 (60.0%)	9 (45.0%)	χ^2^ = 0.94	0.626
Female	9 (45.0%)	8 (40.0%)	11 (55.0%)		
Length	10.24 ± 2.21	10.61 ± 1.96	10.99 ± 2.31	F = 0.60	0.552
Proximodistal width	5.03 ± .67	5.23 ± 1.08	5.43 ± .97	F = 0.94	0.397
Buccolingual width	4.77 ± .61	5.23 ± .98	5.22 ± .78	F = 2.11	0.131
Distance from nasal cavity	3.53 (1.09, 8.50)	7.73 (5.44, 13.15)	6.61 (1.87, 9.05)	χ^2^ = 3.44	0.178
Distance to adjacent teeth	1.24 ± .45	1.43 ± .50	1.33 ± .64	F = 0.67	0.516
*Obstruction type*					
Inverted	7 (29.2%)	8 (33.3%)	9 (37.5%)	χ^2^ = 1.09	0.982
Horizontal	5 (41.7%)	4 (33.3%)	3 (25.0%)		
Oblique	5 (35.7%)	5 (35.7%)	4 (28.6%)		
Orthodontic	3 (30.0%)	3 (30.0%)	4 (40.0%)		
*Located in adjacent teeth*					
Root tip	8 (40.0%)	6 (30.0%)	6 (30.0%)	χ^2^ = 1.20	0.878
Middle segments	7 (35.0%)	7 (35.0%)	6 (30.0%)		
Crown segments	5 (25.0%)	7 (35.0%)	8 (40.0%)		

### Subjects

Patients included in this study met the following criteria:

1. Preoperative CBCT examination to determine that the teeth were osseous-embedded supernumerary teeth;
2. Embedded teeth affecting normal eruption of permanent teeth;
3. Orthodontic treatment requiring extraction or causing odontogenic cysts;
4. No systemic diseases and contraindications to tooth extraction.

Patients with the following conditions were excluded:

1. Have sprouted extra teeth;
2. Have contraindications of anesthesia;
3. Pregnant or lactating women;
4. Patients are unable to cooperate due to mental illness or other reasons, with poor compliance

### Surgical technique

#### Instruments

CBCT imaging system and image processing software (GalileosViewer, observation accuracy: < 10 μm, positioning accuracy: < 100 μm, senora, Germany); High-frequency electric knife (GD350-B, China). Shanghai), Computer-controlled Oral anaesthesia (STA, USA), 45° elevation angle surgical extraction turbine, long fracture drill for tooth extraction, minimally invasive tooth extraction surgical instrument kit and other Surgical extraction instruments, etc.

#### Procedure

**Fig. 2:**
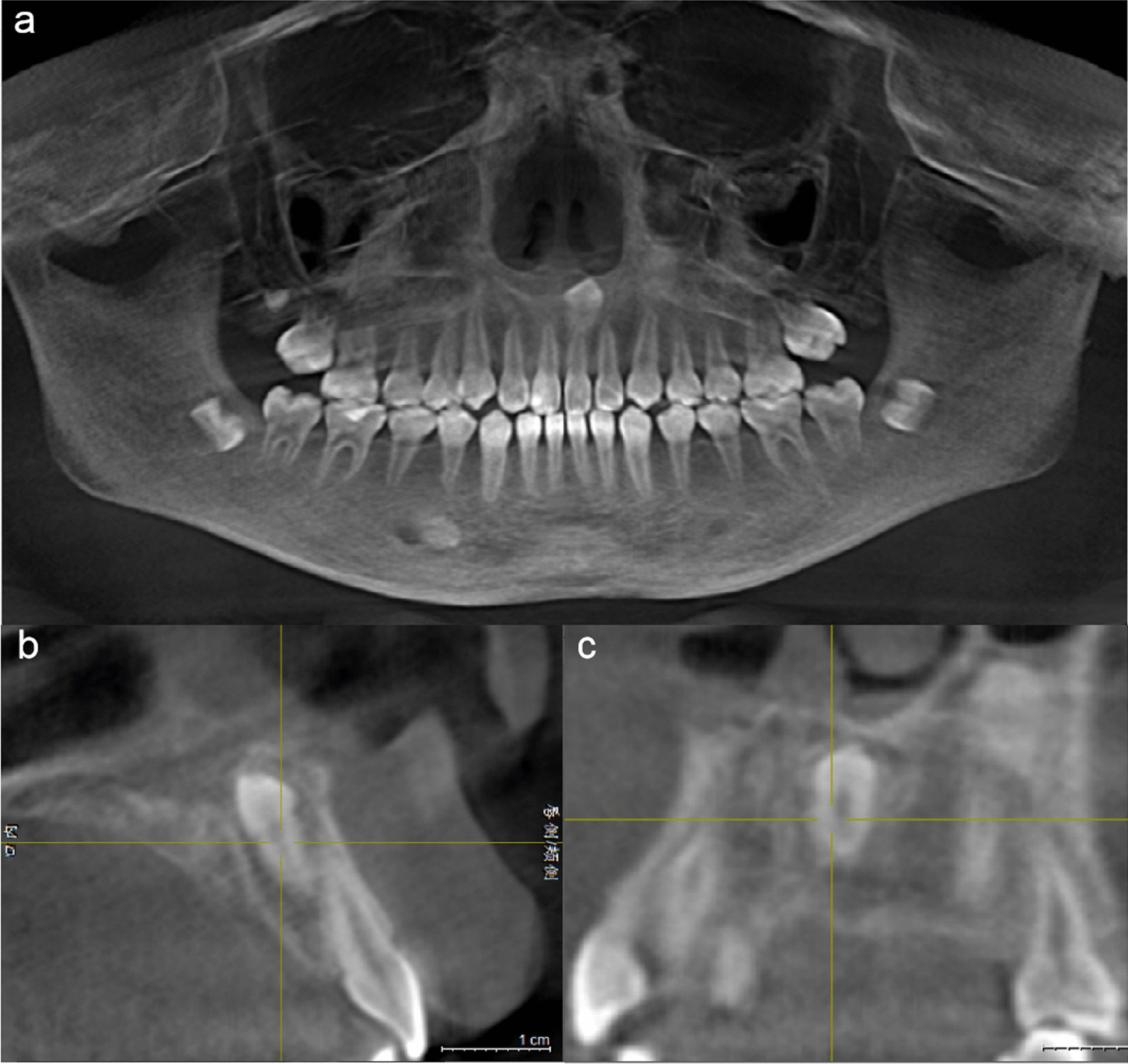
Pre-operative CBCT data of the patient. **a** Preoperative CBCT picture shows that: an inverted embedded supernumerary teeth can be seen above the 21 apex, close to the nasal cavity; **b** Crown position; **c** Horizontal position

**Fig. 3:**
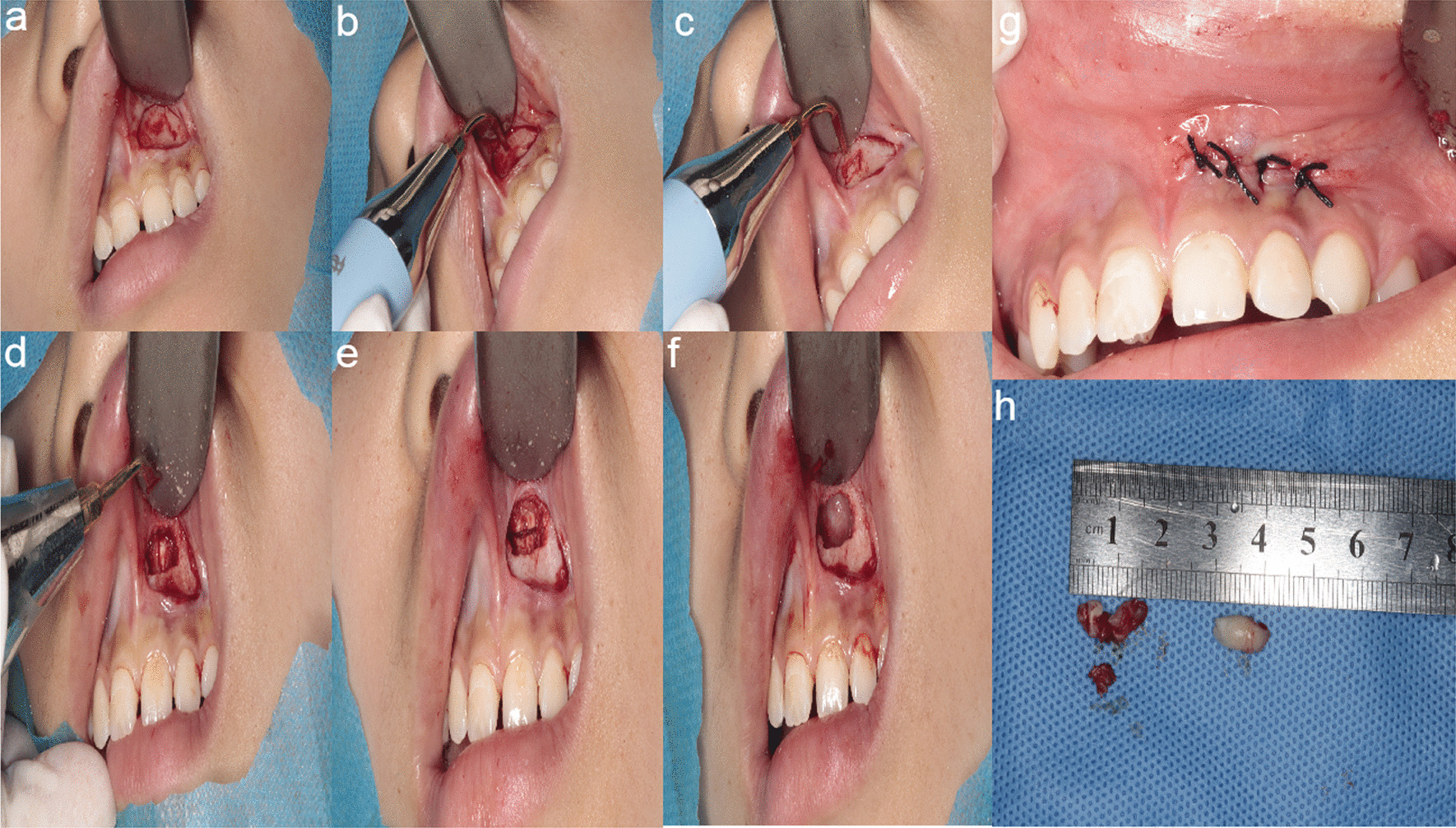
21 Procedure for extraction of palatal lateral embedded supernumerary teeth. **a** Flap straight to the bone surface; **b**, **c** debridement, correction of the open surface; **d**, **e** split the tooth, remove the crown and root in pieces; **f** cleaning the alveolar sockets; **g** Suture; **h** Extracted embedded supernumerary teeth

Pre-operative cone beam CT was used to determine the location, orientation, and relationship to adjacent roots and anatomical structures such as nerves and blood vessels, as well as the closest point to the bone surface and analyze the resistance, and then develop the surgical plan (Fig. 2a–c). After routine disinfection and towel laying, local anesthesia was applied and after the anesthesia took effect, the surgical incision was made according to the design, and the bone surface was exposed. piezosurgery combined with HSCAH group: use piezosurgery to debone the window, reveal the embedded teeth; replace the working tip, increase the gap around the embedded teeth, if needed to divide the tooth, use the HSCAH to divide the tooth, then use a tooth brace or scraper to remove the embedded teeth. HSCAH group: use the turbine contra-angle handpiece to debone the window, reveal the embedded teeth if it is necessary to divide the teeth, use the turbine to divide the teeth, and then remove the embedded teeth with instruments such as dental jaws or scrapers. Saline rinses the alveolar sockets. Wait for fresh blood to fill the extraction sockets, reset the mucoperiosteal flap and suture the gingival tissue in place (Fig. 3a–h). local pressure to stop bleeding for 30 ~ 40 min after surgery and explain the precautions to be taken after tooth extraction The patient was instructed to follow up on the 1st, 3rd, 5th, and 7th postoperative days, and the stitches were removed after 7 d. The patient was given oral analgesics for 1 d and antibiotics for 3–5 d.

#### Evaluation parameters

1. Operative time (time from incision to the end of suturing);
2. intraoperative bleeding Blood loss = (weight of blood gauze a weight of dry gauze) + the amount of blood in the suction bottle will be weighed before the operation, to use dry gauze instead of saline gauze during the operation, the amount of blood in the suction bottle should be noted minus the possible amount of saline or other liquids, the unit of weight is "g", 1 ml of blood is calculated as 1 g.;
3. Postoperative the changes in pain were observed in the three groups at 1d, 3d, and 7d postoperatively and were assessed using the Visual Analogue Scale (VAS) for pain, with a score of 10, the higher the score the more severe the pain;
4. Swelling Assessment was done by measurements, which were performed for the same person and averaged after three measurements. Specifically, the patient's pre-operative and post-operative distances were measured at 24 and 72 h (distance between the angle of the jaw and the corner of the mouth, the distance between the angle of the jaw and the nose, and distance between the angle of the jaw and the external canthus). Then the sum of the postoperative measured distances was subtracted from the sum of the preoperatively measured distances, and the degree of postoperative swelling would be classified into four categories according to the values obtained. The value obtained ≤ 1 indicates normal; the value obtained 1–3 is mild swelling; the value obtained 3–5 is moderate swelling; and the value obtained > 5 indicates severe swelling.
5. Dental phobia was assessed using a questionnaire: very afraid: 1 point, somewhat afraid: 2 points, not feeling: 3 points, 4 without any fear: 4 points.

### Statistical analysis

SPSS 23.0 statistical software was applied for data collation and processing, and multiple groups were analyzed by ANOVA and LSD for two-way comparisons if they obeyed chi-squareness, and by median and interrogative spacing if they did not, and by rank sum test. The statistical data were expressed as percentages (%) using the χ^2^ test. *p* < 0.05 was considered a statistically significant difference.

## Results

**Fig. 4:**
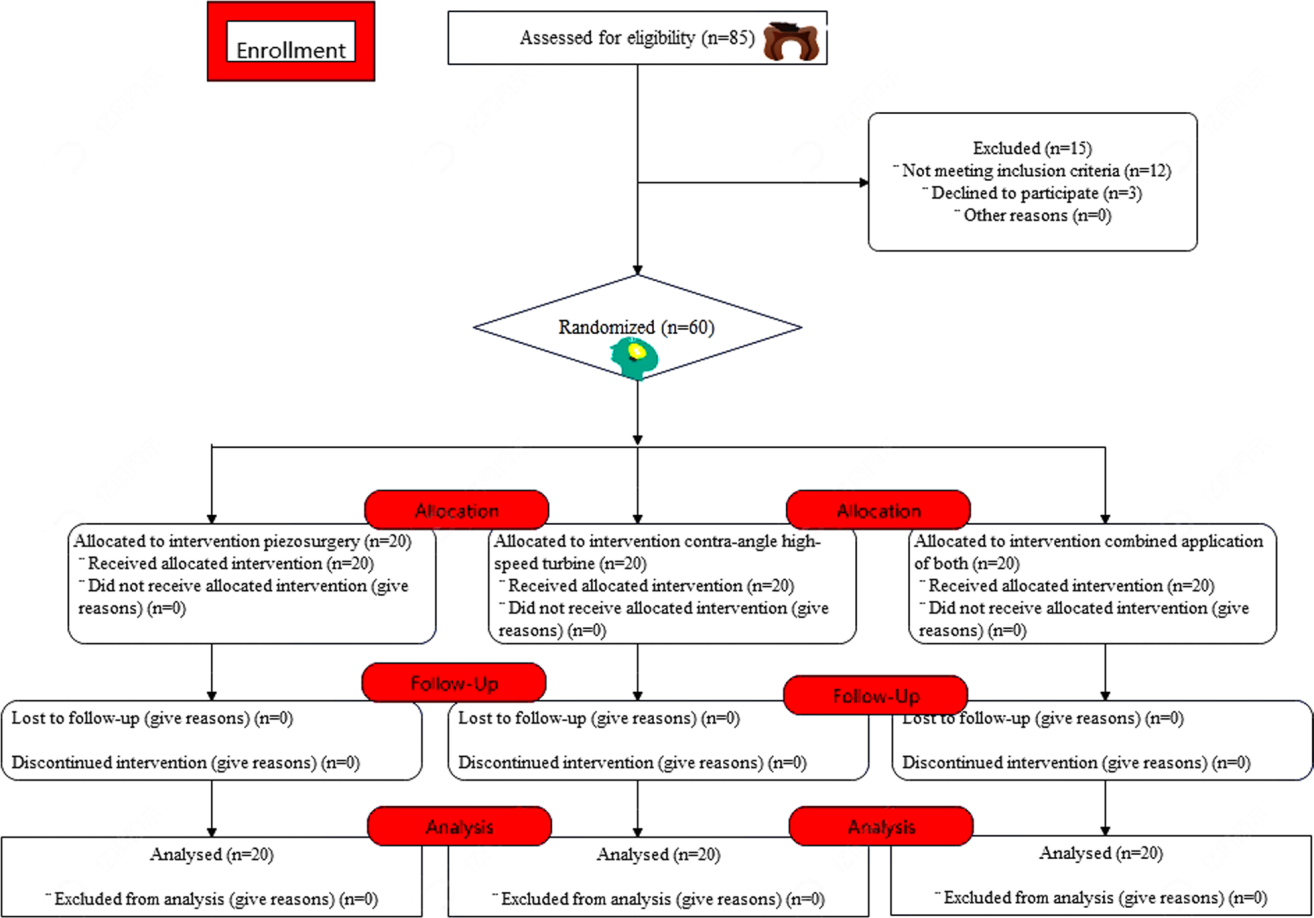
Flow chart of patient selection

The flow chart of this study is presented in Fig. 4.

*General information situation* For a description of the patients' general data, see (Table 1).

### Operating time

**Fig. 5:**
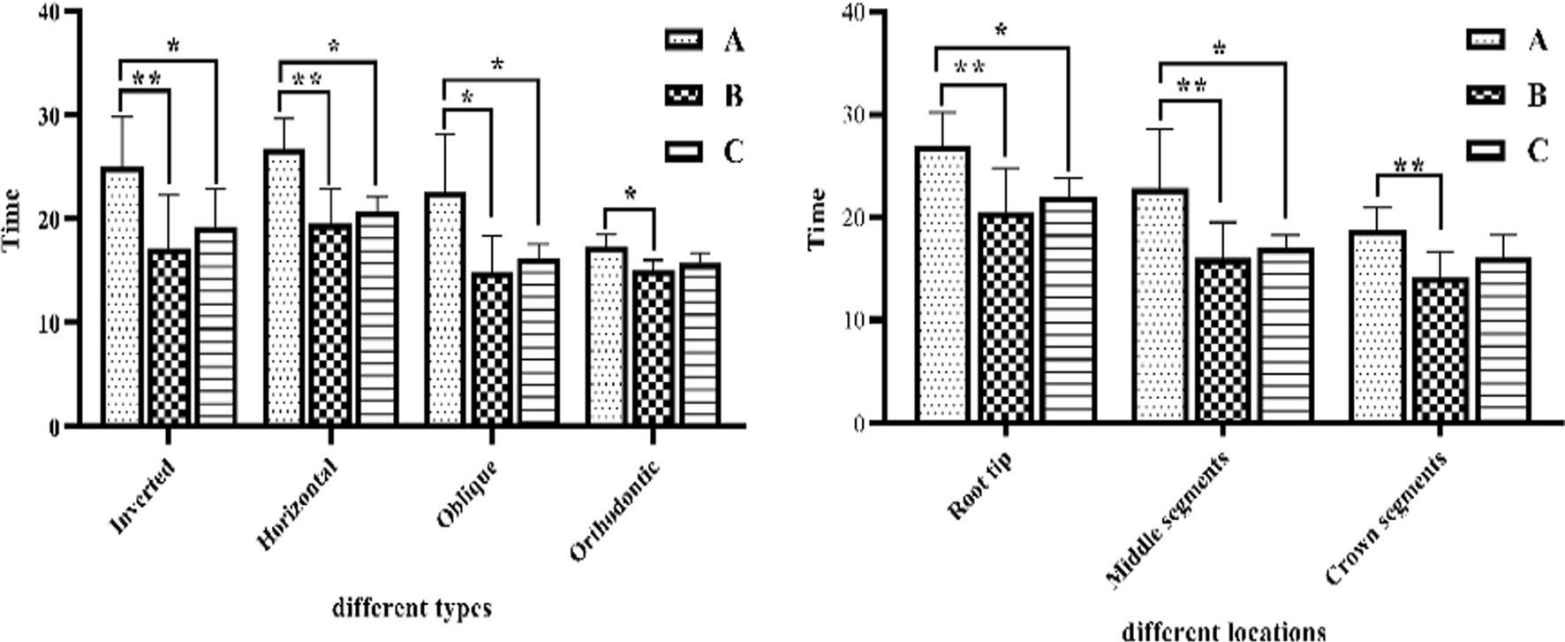
Comparison of surgical time for extraction of different locations and types of embedded supernumerary teeth; **A** piezosurgery; **B** HSCAH; **C** Combined application of both

Comparison of the operative times of different buried polypoid teeth revealed significant differences in both extractions of the inverted and horizontal aspects in the piezosurgery group compared to the HSCAH group (*p* < 0.01) and statistically different compared to the combined piezosurgery group (*p* < 0.05). For extraction of oblique position, there was a statistical difference (*p* < 0.05) between the piezosurgery group and the HSCAH and combined piezosurgery groups; for extraction of orthotropic position, there was a statistical difference (*p* < 0.05) between the piezosurgery and HSCAH groups. Comparison of operative times for different ambulation locations showed that there was a statistically significant difference (*p* < 0.01) between the piezosurgery group and the HSCAH group in the extraction of both Root tip and middle segments, compared to the combined piezosurgery group (*p* < 0.05). There was a significant difference between the piezosurgery group and the HSCAH group in terms of extraction of the crown segment (*p* < 0.01). See Fig. 5.

### Operative bleeding

**Fig. 6:**
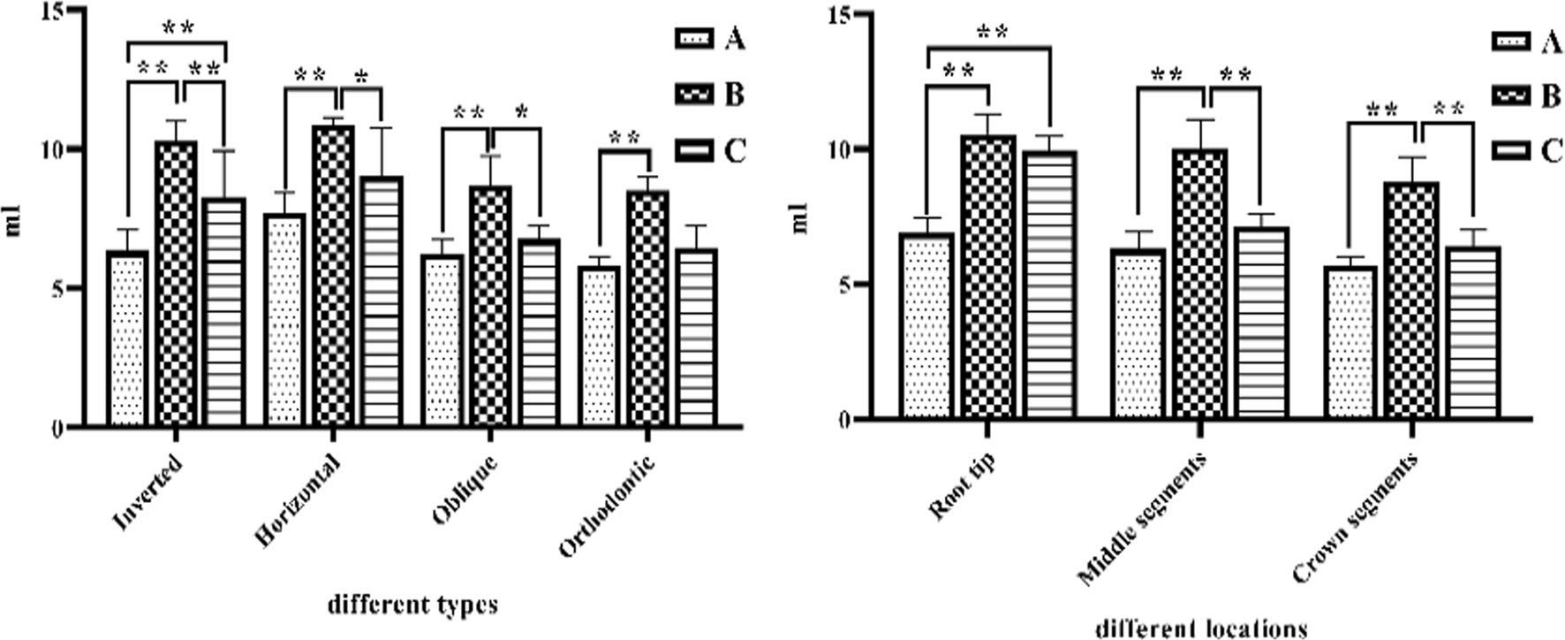
Comparison of bleeding volume in extracting different locations and types of embedded supernumerary teeth; **A** piezosurgery; **B** HSCAH; **C** Combined application of both

Comparison of intraoperative bleeding in different embedded supernumerary teeth revealed a significant difference between the piezosurgery group, the HSCAH group, and the combined piezosurgery group in the extraction of inverted embedded supernumerary teeth (*p* < 0.01). A significant difference between the piezosurgery group compared to the HSCAH group in the extraction of horizontally and obliquely positioned embedded supernumerary teeth (*p* < 0.01); the statistically significant difference between the HSCAH group compared to the combined piezosurgery group (*p* < 0.05).A significant difference between the piezosurgery group and the HSCAH group in the extraction of orthodontically embedded teeth (*p* < 0.01). Comparison of bleeding in different ambulant locations showed that there was a significant difference (*p* < 0.01) between the piezosurgery group compared with the HSCAH group and the combined piezosurgery group in the extraction of embedded supernumerary teeth with root tips. There was a significant difference (*p* < 0.01) between the piezosurgery group compared with the HSCAH group in the extraction of the middle and crown segments of the embedded supernumerary teeth; there was a significant difference (*p* < 0.01) between the HSCAH group compared with the combined piezosurgery group. See Fig. 6.

### Postoperative pain

**Fig. 7:**
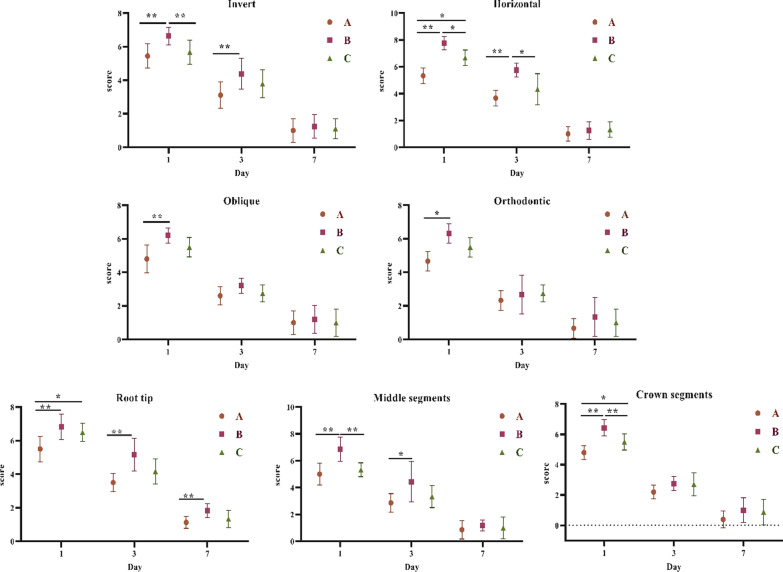
Comparison of VAS scores for extraction of different positions and types of embedded supernumerary teeth; **A** piezosurgery; **B** HSCAH; **C** Combined application of both

The results of the comparison of VAS scores of different ambulatory types showed that on day 1 after extraction of inverted, horizontally embedded supernumerary teeth, there was a significant difference between the piezosurgery, HSCAH, and combined piezosurgery groups (*p* < 0.01); on day 3 after surgery, there was a significant difference between the piezosurgery and HSCAH groups (*p* < 0.05); in the HSCAH and combined piezosurgery groups comparison, there was a significant difference (*p* < 0.05). There was no significant difference between the three groups at postoperative day 7 (*p* > 0.05). There was a significant difference (*p* < 0.05) between the piezosurgery and the HSCAH on postoperative day 1 after extraction of oblique and orthodontic embedded supernumerary teeth; there was no significant difference (*p* < 0.05) between the three groups on postoperative days 3 and 7. The results of the comparison of VAS scores in different ambulatory positions showed that there was a significant difference (*p* < 0.05) between piezosurgery and HSCAH on the 1st day after extraction of embedded teeth in oblique and orthodontic positions, and no significant difference (*p* < 0.05) on the 3rd and 7th day after surgery. A significant difference (*p* < 0.01) between piezosurgery and HSCAH on postoperative day 1 after extraction of embedded supernumerary teeth at the middle and crown segments; no significant difference (*p* > 0.05) between the three groups on postoperative day 3 compared to day 7. See Fig. 7.

### Swelling

**Fig. 8:**
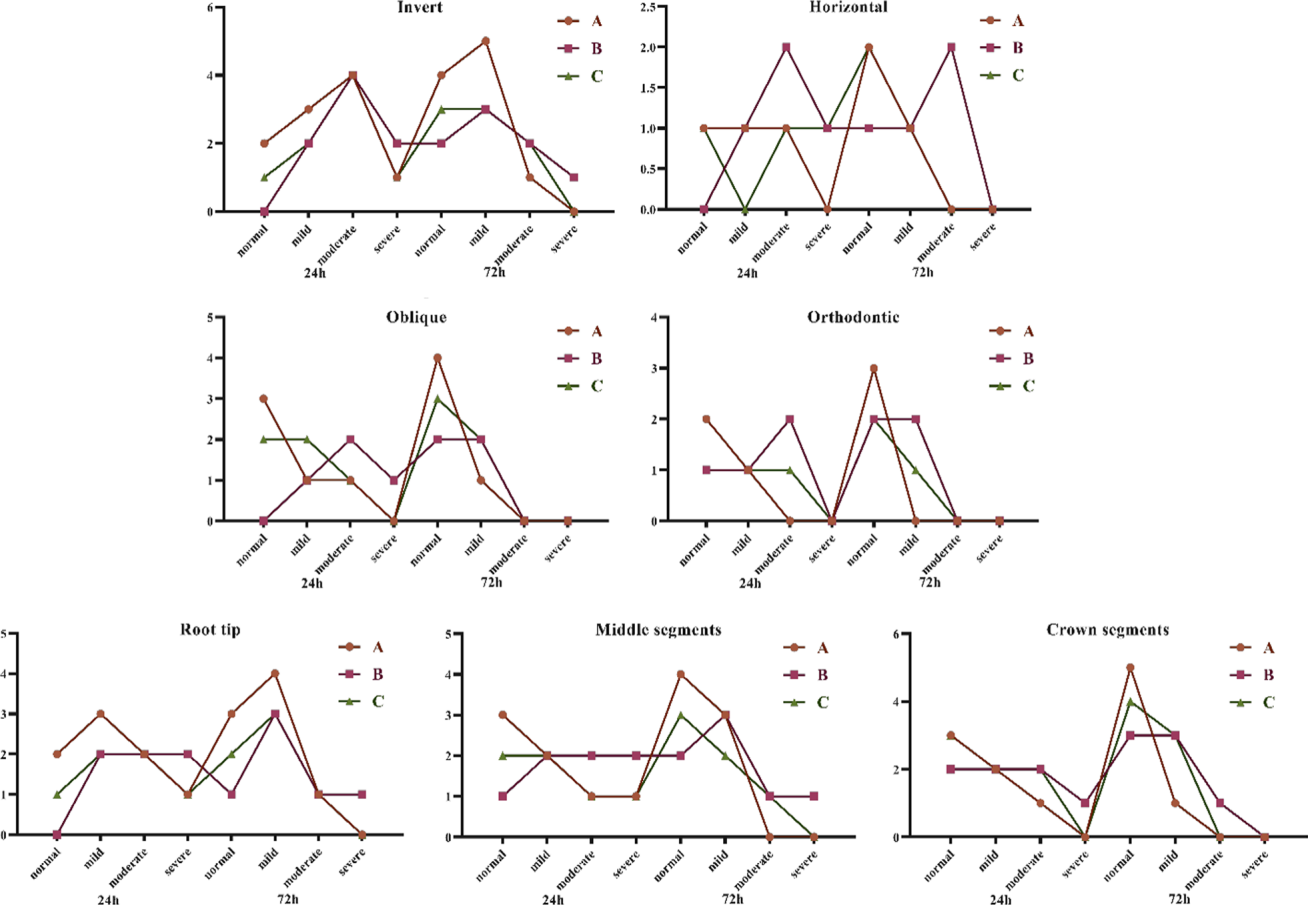
Comparison of facial swelling in the extraction of different positions and types of embedded supernumerary teeth; **A** piezosurgery; **B** HSCAH; **C** Combined application of both

Comparison of facial swelling in different ambulant types showed that the normal incidence of swelling at 24 h and 72 h after treatment was significantly higher in the piezosurgery and combined piezosurgery groups than in the HSCAH group, while the incidence of severe swelling was significantly lower in the extraction of inverted and horizontally embedded supernumerary teeth. In the extraction of oblique and orthodontic embedded supernumerary teeth, the normal incidence of swelling at 24 h postoperatively was higher in the piezosurgery group than in the HSCAH group, and the normal incidence of swelling at 72 h postoperatively was significantly higher in three groups, whereas the incidence of severe was significantly lower. Comparison of facial swelling in different ambulatory locations revealed that in embedded supernumerary teeth with root tips extracted, the normal incidence of swelling was higher in the piezosurgery group than in the HSCAH group at 24 h postoperatively, while the proportion of patients with severe incidence was lower than in the HSCAH group, and the normal incidence was significantly higher and the severe incidence was significantly lower in all three groups at 72 h postoperatively. In the middle and crown segments of the embedded supernumerary teeth, the normal incidence was significantly increased in all three groups at 24 h postoperatively, while the incidence of severe was significantly decreased. See Fig. 8.

### Phobia

**Fig. 9:**
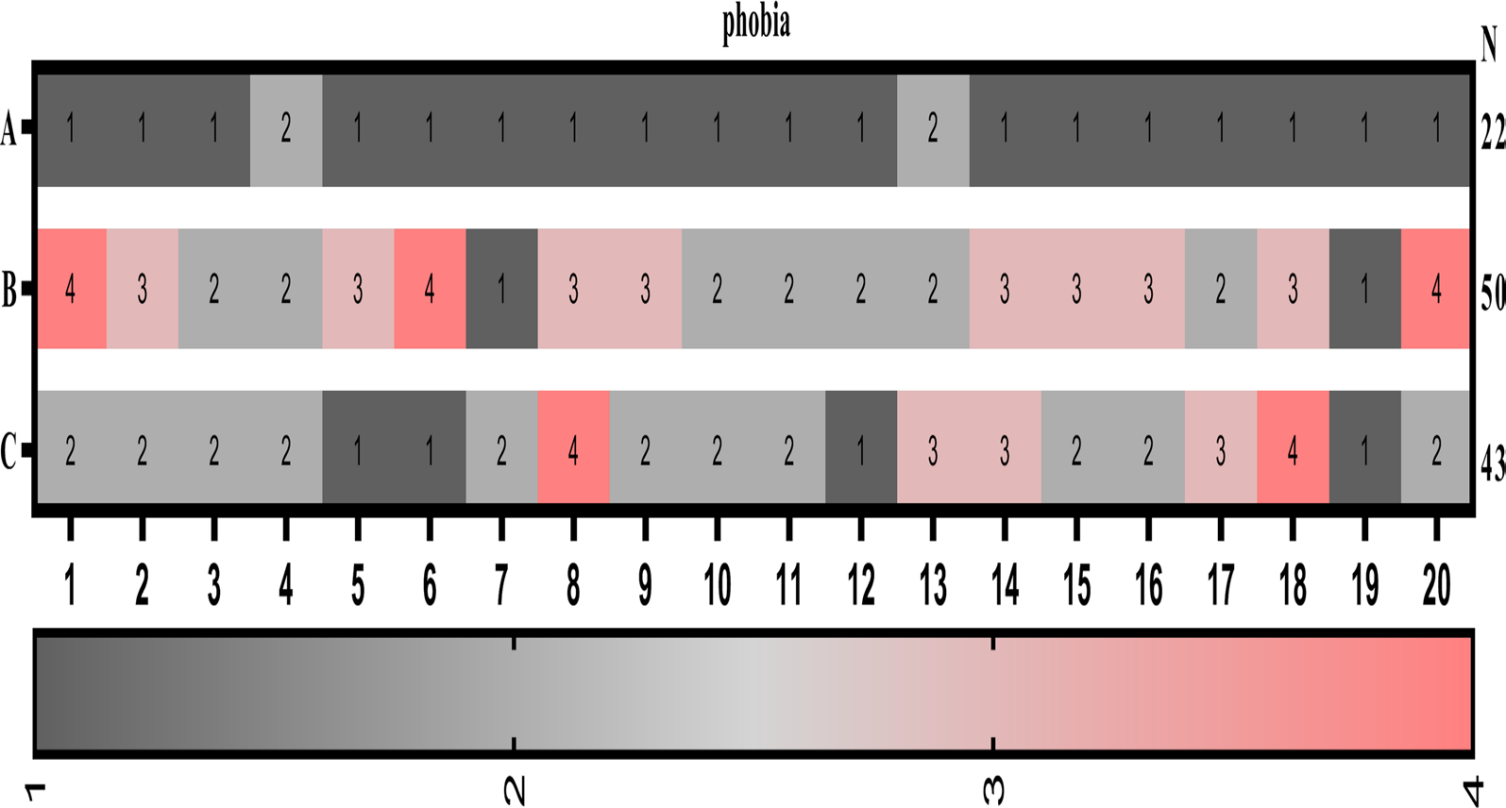
Comparison of phobias across treatment options; **A** piezosurgery; **B** HSCAH; **C** Combined application of both

The incidence of post-extraction fear was significantly lower in the piezosurgery compared to the HSCAH, combined piezosurgery. See Fig. 9.

### Fi-index tool

This manuscript has been checked for all authors with fi-index Tool with a score of 0.10 for the corresponding author in date 16/1/2023 according to Scopus (Table 2).

**Table 2:** Fi-index tool score for author detail table

Author name	Fi-index tool score
Maihepireti Maihemaiti	0
Ailimaierdan Ainiwar	0.11
Ling Wang	0.10

## Discussion

In this clinical study, we collected 60 patients who needed to have their embedded supernumerary teeth extracted. In this study, we classified the embedded supernumerary teeth by the impacted data of the patients according to the type of obstruction (inverted, horizontal, oblique, orthodontic) and the position about the long axis of the adjacent teeth (root tip, middle, and crown segments). Three extraction methods were performed on different locations and types of buried multiple teeth, and their surgical results were evaluated. The piezosurgery group required the longest operative time for the extraction of inverted, horizontal, oblique, and root tip, middle embedded supernumerary teeth, but had the least postoperative bleeding, was effective in reducing postoperative pain and swelling, and significantly reduced patient fear compared to the HSCAH and combined piezosurgery. When extracting inverted, horizontal, oblique, and root tip, and middle embedded supernumerary teeth, the HSCAH, and combined piezosurgery groups can significantly reduce the operative time; however, in the HSCAH group, the bleeding is higher and the postoperative pain and swelling are significant.

In this study, we evaluated the surgical outcomes of different locations and types of embedded supernumerary teeth with piezosurgery, HSCAH, and combined piezosurgery, and assessed the operative time, intraoperative bleeding, and two postoperative variables, namely pain, and facial swelling, and a fourth variable, phobia of psycho-emotional reaction.

In terms of operative time, in a study by Jiang [11] and others, it was concluded that the operative time was longer with piezosurgery compared to the use of a high-speed turbine, because the speed of bone cutting was slower. Mantovani et al. [12], Mistry [13], and other studies have concluded that HSCAH, which can cut bone quickly, can significantly reduce the operation time. In our study, the operative time of the high-speed turbine group was faster than either the piezosurgery group or the combined piezosurgery group in different positions (inverted, horizontal, oblique) and types (root tip, middle) of extractions of embedded supernumerary teeth, which is consistent with the results of other studies and the combined application of HSCAH with piezosurgery can compensate for the slow speed of piezosurgery osteotomy.

In terms of the amount of surgical bleeding, Stbinger et al. [14], concluded that the use of piezosurgery can significantly reduce the amount of surgical bleeding during surgery. Similar conclusions were reached in case reports such as Sukegawa [15]. In our study, the use of piezosurgery significantly reduced bleeding in inverted, horizontal, and root-tip embedded supernumerary teeth, and bleeding was significantly less with the application of piezosurgery and combined piezosurgery for oblique, orthodontic, middle, and coronal embedded supernumerary teeth compared with the HSCAH group. The reason for this is that the cavitation effect in saline acts to form a bubble in the body and implodes into the blood vessels to produce shock waves that cause micro coagulation in the blood vessels to act as hemostasis. Thus reducing bleeding [16].

The piezosurgery operates at 24.0 to 29.5 kHz and can selectively perform cutting functions, using different operating frequencies for soft and hard tissues, cutting only mineralized structures-bone, but stopping when it encounters nerve, blood vessels, or other soft tissues. Less invasive damage during cutting, less damage to surrounding tissues, and promotes tissue repair. The release of oxygen ions during cutting provides a certain antiseptic effect and does not cause bone necrosis near the cutting area. When ultrasound works it stimulates cells and promotes tissue metabolism and bone regeneration. When an HSCAH performs osteotomy, a smooth surface is formed around the bone. When an piezosurgery performs osteotomy, an irregular rough surface is formed around the bone, and more osteoclasts and osteoblasts on this rough bone edge work simultaneously to promote bone regeneration. When an HSCAH cuts bone, the working head heats up, which causes thermal damage to bone tissue; however, the piezosurgery cutter head generates less heat, so it can prevent thermal damage to bone tissue during osteotomy [17–20]. Because of the above-mentioned advantages of piezosurgery, healing after the procedure is faster than with HSCAH, and it can greatly reduce postoperative pain and swelling compared to HSCAH.

In terms of the level of pain being assessed by VAS, in a study by Topcu [21] and other investigators, it was found that the mean VAS scores in the piezosurgery group intraoperatively, on the second postoperative day, were significantly lower than those in the HSCAH group, while the difference in pain scores between the piezosurgery group and the HSCAH group on the seventh postoperative day was not statistically significant. Maglione [22] and other researchers concluded that in the piezosurgery, all VAS pain scores were significantly lower than in the HSCAH group 1–5 days after surgery. In our study, although the procedure was time-consuming, the application of piezosurgery and combined piezosurgery for extraction of inverted, horizontal, and root tip buried multiple teeth on postoperative days 1 and 3 resulted in lower VAS scores than the HSCAH group, and we followed up until postoperative day 7 with improvement in pain in all three groups, indicating a recovery in all three groups, yielding the same results. The application of piezosurgery and combined piezosurgery for extraction of the oblique, orthodontic, middle, and coronal segments of embedded supernumerary teeth showed a faster decrease in VAS scores on a postoperative day 1 than the HSCAH group, and no significant difference was seen between the three groups at follow-up until day 3, indicating significant postoperative pain relief in all three groups.

In terms of facial swelling, Mistry [13] and other researchers concluded that swelling was significantly worse in the HSCAH group than in the piezosurgery group on postoperative days 1, 3, 5, and 7. Shahakbari [23] and other researchers found that the rate of reduction in measurements based on facial swelling was significantly higher in the piezosurgery group than in the HSCAH group at 7 days postoperatively. In our study, piezosurgery and combined piezosurgery applied for extraction of inverted, horizontal, and root tip embedded supernumerary teeth showed faster swelling reduction at 24 h and 72 h postoperatively, i.e., higher normal incidence than in the HSCAH group. The incidence of a normal 24-h postoperative period was significantly increased in all three groups for the extraction of oblique, orthodontic, middle, and coronal segments of embedded supernumerary teeth, while the incidence of severe was significantly decreased.

For postoperative fears, there have been many studies on the extraction of obstructed wisdom teeth [24–26], but there are few reports on the extraction of embedded supernumerary teeth. In psychology, anxiety and fear are psychological reactions to uncomfortable or unpleasant stimuli. Studies have shown [27, 28] that patients' anxiety or fear is related to age, gender, literacy, and personality. This negative psychological response is prevalent in dentistry and it is the fourth most prevalent among other psychological disorders [29]. In clinical treatment, the main psychological reactions of patients are fear, anxiety, stress, and pain sensitivity, and, during treatment, the psychological and emotional state of the patient is very important for the comfort of both the doctor and the patient [30]. High patient anxiety inevitably complicates dental treatment and also affects patient compliance and comprehension during diagnosis and treatment. According to the study [31], not all dental treatments cause dental phobia, and one of the most potent dental treatments that cause dental phobia is extraction. In other studies, it was concluded [32] that if patients have a high level of dental anxiety. It makes the extraction more difficult and the procedure takes longer. Therefore, high levels of surgical pain and postoperative pain are often experienced, and these can adversely affect postoperative physiological recovery and can even adversely affect access to care, which may lead to patient avoidance of treatment. In a study by Astramskaitė et al. [33] it was concluded that for patients undergoing wisdom tooth excision and extraction, there was a correlation between an increase in mean scores on the fear and anxiety scale and an increase in the level of pain after the procedure. In the study by Muglali [34] and others, among the causes of preoperative and postoperative anxiety, we found that the difficulty of the procedure does not have an impact on the immediate anxiety of the patient, but there are some underlying causes in the follow-up. When an HSCAH is used for bone removal, the speed of the procedure is too fast to control the extent and direction of the procedure, which leads to tissue damage and increases postoperative pain, thus prolonging the recovery time of the procedure [35]. Moreover, the HSCAH makes a lot of noise, so this can increase the fear of the patient [36]. The piezosurgery generates low wrist pressure, the operator's sensitivity and control over the procedure are greatly improved, and the micro-vibrations generated allow the operator to have a better sense of cutting and precision, thus improving the accuracy of the cut. In addition, the volume generated during operation is lower than that of an HSCAH, thus providing a better medical experience and comfort to the patient [37].In our comparative study of postoperative phobia, we found that the incidence of postoperative phobia was significantly lower in the piezosurgery group compared to the HSCAH and combined piezosurgery groups, while the HSCAH group was able to significantly increase postoperative phobia in patients.

Strengths of the present study include the high-quality study design, the randomization, the similarities of the baseline characteristics across the three groups, and the use of a well-validated instrument, We found that in past studies, both piezosurgery and HSCAHs alone were used for tooth extraction, but the two were not used in combination, and in addition, only the two methods were used to compare and contrast the clinical outcomes after extraction. In this study, we divided the study population into three groups and applied both a piezosurgery and an HSCAH to one group of patients for clinical observation and treatment. And the piezosurgery combined with the HSCAH was used on one group of patients to observe the results of the procedure. Furthermore, In this study, we used CBCT data to classify embedded supernumerary teeth according to the type of obstruction (inverted, horizontal, oblique, orthodontic) and the position about the long axis of adjacent teeth (root tip, middle, and coronal segments). In previous investigations, either imaging data were used to classify the embedded supernumerary teeth or extraction measures were used to compare the clinical outcome after extraction without any classification. The limitation of this study is the relatively small sample size. In this study, only short-term follow-up was used to observe the healing of the extraction sockets when comparing the healing of the three methods of extracting supernumerary teeth, while longer follow-up is needed to determine the healing of the extraction sockets and surrounding hard tissues.

## Conclusion

Using piezosurgery to remove the inverted, horizontal, and root tip embedded supernumerary teeth, although the long operation time is its shortcoming, it can reduce the amount of intraoperative bleeding, postoperative pain, and facial swelling, improve the comfort of patients, and reduce the risk of surgery. Therefore, we recommend that the application of piezosurgery be considered first in this case. However, the application of piezosurgery combined with HSCAH is first considered in the extraction of oblique, orthodontic, middle and crown segments of embedded supernumerary teeth.

## Data Availability

The datasets generated and/or analyzed during the current study are not publicly available due to privacy and ethical concerns but are available from the corresponding author on reasonable request.

## Abbreviations

HSCAH: High-speed contra-angle handpiece
CBCT: Cone beam computed tomography
VAS: Visual analogue scale
ANOVA: Analysis of variance
LSD: Least significant difference
